# P-140. Incidence of Heart Failure and Valvular Diseases After Infective Endocarditis: A Propensity-Matched Cohort Study

**DOI:** 10.1093/ofid/ofaf695.367

**Published:** 2026-01-11

**Authors:** Siddartha Guru, Paddy Ssentongo, Nadim Jaafar, Chen Song

**Affiliations:** Penn State Health Milton S. Hershey Medical Center, Hummelstown, PA; Penn State Health Milton S. Hershey Medical Center, Hummelstown, PA; Greater Baltimore Medical Center, Towson, Maryland; Penn State Hershey Medical Center, Hershey, Pennsylvania

## Abstract

**Background:**

Infective endocarditis (IE) is a known risk factor for cardiac remodeling, but population-level data on long-term incidence of heart failure (HF) and valvular disease following IE remain limited.

**Figure 1.**
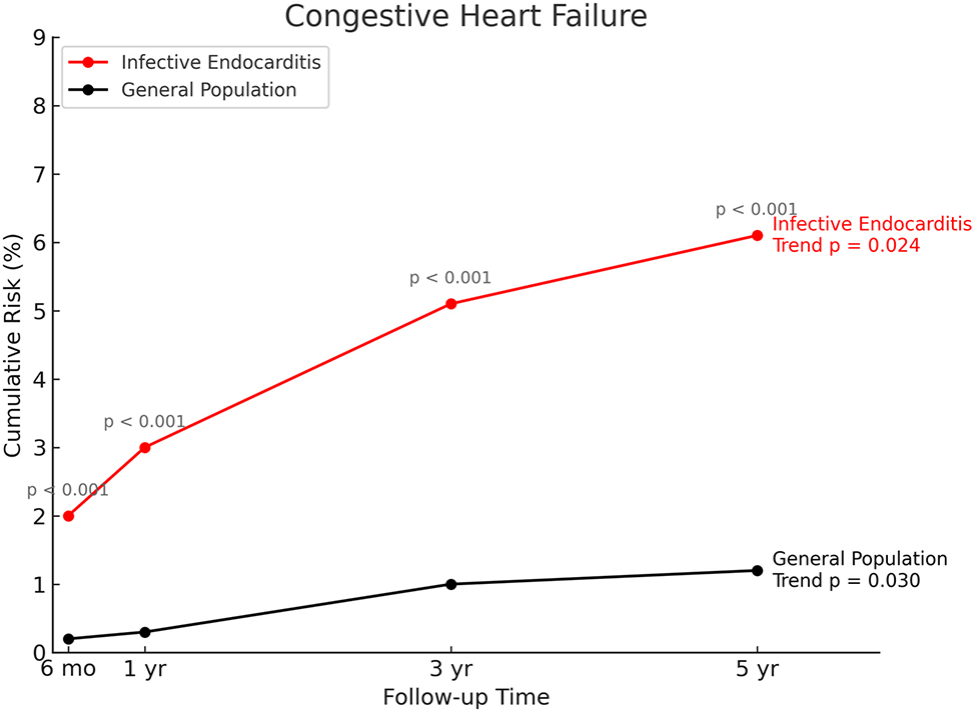
Cumulative incidence of heart failure (HF) in patients with infective endocarditis versus matched general population controls over 5 years of follow-up, beginning 2 weeks after the index event. The incidence of CHF was significantly higher in the infective endocarditis group at 6 months, 1 year, 3 years, and 5 years post-index (all P < .001). Trend analyses showed a sustained increase in cumulative risk in both groups (IE trend P = 0.024; general population trend P = 0.030), with early divergence evident by 6 months.

**Figure 2:**
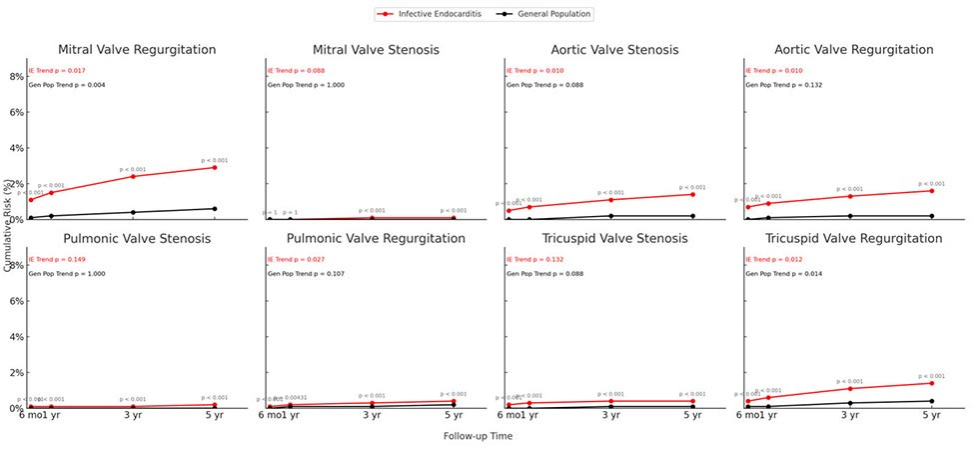
Cumulative incidence of valvular diseases in patients with infective endocarditis (IE) versus matched general population controls over 5 years of follow-up, beginning 2 weeks after the index event. Across all valve types, patients with IE had significantly higher cumulative incidence of regurgitation and stenosis compared to controls (all P < .001 unless otherwise indicated). Mitral and aortic valve involvement showed the highest absolute risks, while right-sided and pulmonic valve outcomes were less frequent. Trend analyses indicated statistically significant increases over time for several valve pathologies in the IE group, particularly mitral regurgitation (P = 0.017) and aortic valve lesions (P = 0.010).

**Methods:**

We conducted a retrospective cohort study using the TriNetX Global Collaborative Network. Patients aged ≥18 years with a new diagnosis of infective endocarditis and no prior history of heart failure or structural valvular disease were matched via 1:1 propensity score to a general population cohort without IE or heart failure. Matching was performed on demographic and comorbidity variables. The primary outcome was the incidence of HF at 6 months, 1 year, 3 years, and 5 years post-index. Secondary outcomes included mitral, aortic, tricuspid, and pulmonic valve regurgitation and stenosis. All outcomes were measured beginning 14 days after the index diagnosis to exclude preexisting conditions.

**Results:**

Compared to the general population, the IE cohort had significantly higher 5-year cumulative incidence of HF (6.1% vs 1.2%; hazard ratio [HR], 6.92; 95% CI, 6.14–7.80, Figure 1), mitral regurgitation (2.9% vs 0.6%; HR, 6.53; 95% CI, 5.50–7.76), and aortic regurgitation (1.6% vs 0.2%; HR, 6.41; 95% CI, 4.93–8.33), among other valve outcomes (all P < .001, Figure 2). Time trends for all outcomes were significant across follow-up intervals. Cumulative event curves showed early and sustained divergence between cohorts beginning within the first year (Figure).

**Conclusion:**

Infective endocarditis is associated with a markedly increased risk of incident heart failure and progressive valvular pathology, with differences emerging within the first year and persisting through 5 years. These findings support long-term echocardiographic surveillance and early cardiology follow-up in patients recovering from IE.

**Disclosures:**

All Authors: No reported disclosures

